# Sources, diffusion and prediction in COVID-19 pandemic: lessons learned to face next health emergency

**DOI:** 10.3934/publichealth.2023012

**Published:** 2023-03-02

**Authors:** Mario Coccia

**Affiliations:** National Research Council of Italy, Department of Social Sciences, Turin Research Area of the National Research Council-Strada delle Cacce, 73-10135 - Torino (Italy)

**Keywords:** COVID-19 pandemic, infectious diseases, environmental factors, compartmental models, epidemiologic models, outlook, preparedness, surveillance, public health, health policy, policy responses, crisis management

## Abstract

Scholars and experts argue that future pandemics and/or epidemics are inevitable events, and the problem is not whether they will occur, but when a new health emergency will emerge. In this uncertain scenario, one of the most important questions is an accurate prevention, preparedness and prediction for the next pandemic. The main goal of this study is twofold: first, the clarification of sources and factors that may trigger pandemic threats; second, the examination of prediction models of on-going pandemics, showing pros and cons. Results, based on in-depth systematic review, show the vital role of environmental factors in the spread of Coronavirus Disease 2019 (COVID-19), and many limitations of the epidemiologic models of prediction because of the complex interactions between the new viral agent SARS-CoV-2, environment and society that have generated variants and sub-variants with rapid transmission. The insights here are, whenever possible, to clarify these aspects associated with public health in order to provide lessons learned of health policy that may reduce risks of emergence and diffusion of new pandemics having negative societal impact.

## 1. Introduction

In 2023, negative effects of the Coronavirus Disease 2019 (COVID-19) pandemic are considerably reduced, unlike the early period in 2020, though many people still have to cope with COVID-19 illness [1]–[14]. In the USA, deaths per day of COVID-19 in the early months of 2023 are higher than deaths with a seasonal influenza. These facts suggest that COVID-19, in a different way, continues to have a certain impact in society [15].

A vital aspect for public health and security of nations is to show the lessons learned to face COVID-19 pandemic crisis, to improve the prevention and preparedness, to stop and/or mitigate, whenever possible, the emergence and diffusion of a new virus that infects a lot of people worldwide [16]–[21]. In this context, the goal of this study is a general analysis of the sources and driving factors of Severe Acute Respiratory Syndrome Coronavirus 2 or SARS-CoV-2 virus (leading to COVID-19), and an examination of prediction approaches, showing pros and cons [22]–[27]. The prediction and preparation to deal with a pandemic, effectively, involve different aspects, such as how new viral agents may emerge, behave and mutate in environments, to explain the transmission dynamics, spatial diffusion and impact in society [22],[28]–[30]. In the presence of the COVID-19 pandemic, governments have largely applied epidemiological models of prediction of cases and/or deaths to guide the effective and timely implementation of health policies based on restrictions and/or vast vaccination campaigns [31]–[34]. However, epidemiologic models of COVID-19 have also shown many limitations because of unpredictable behavior of the SARS-CoV-2 virus in the environment and society [35].

Hence, since the source and diffusion of the pandemic are associated with manifold factors and the forecasting of pandemic dynamics, using current epidemiologic models can have shortcomings with mutant viral agents and/or changes in mitigation and containment policies of nations. The analysis and discussion of these aspects can show lessons learned to improve the guidelines in the governance and public health of countries, in a post pandemic period, to avoid the emergence of new outbreaks [36].

## 2. Materials and methods

### 2.1. Databases and search strategy

The systematic review used here is based on a search strategy of selected papers published in various databases, such as PubMed [37], Scopus [38] and Web of Science [39]. The search strategy was formulated and refined in terms of subject keywords by a “Boolean searching” using the operator of logical conjunction AND; some of the search strings used here are: COVID-19 AND sources, COVID-19 AND “diffusive factors”, COVID-19 AND “prediction models”, etc. Quotation marks were used to search in a loose phrase, where the words appear together in a fixed order.

### 2.2. Inclusion and exclusion criteria

Current search engines, with the above strings, provide a high number of papers, including many irrelevant resources. Therefore, for effective analyses, the study here follows a systematic search strategy that screens and selects the literature to find the relevant papers based on specific criteria. In this study, three main inclusion and exclusion criteria are applied for identifying relevant content and removing irrelevant papers (Table 1) [40]–[42]:

a) The first inclusion criterion is the type of document; published documents are included, whereas manuscripts under review and unpublished manuscripts are excluded.

b) The domain (i.e., the subject area identified for the study) is the second screening criterion; papers dealing strictly with factors of emergence and diffusion of COVID-19, and about prediction models for COVID-19 are included, whereas other documents are excluded.

c) The last screening criterion is the language in which the paper is published; inclusion criterion is English; exclusion is non-English language.

**Table 1.** Inclusion and exclusion criteria of papers of the study.

Criterion	Inclusion	Exclusion
Document type	Published papers	*Unpublished* manuscripts
Scientific domain	Papers dealing with factors of emergence and diffusion of COVID-19, and about prediction models for COVID-19	Papers *non* dealing strictly with factors of emergence and diffusion of COVID-19, and about prediction models for COVID-19
Language	English papers	*Non*-English papers

### 2.3. Eligibility and inclusion for review and qualitative synthesis of results

Quality evaluation is conducted to avoid biases and errors. In this study, in the initial phase, more than 620 documents were chosen. After an in-depth analysis, using criteria of Table 1, records screened and selected are 62 documents that are also the papers assessed for eligibility in this study (10% of the total). Papers excluded for the lack of information strictly related to the topics under study here are 31 documents. Hence, papers selected for the review and the analysis for a qualitive synthesis of results are 31 (50% of record selected).

In this perspective, systematic review here focuses on a method of logic selection of specific literature (above) aimed at minimizing bias in order to produce reliable findings that explain drivers of new viral agents, factors related to diffusion and societal impact, and points of strength and weakness in epidemiological models [43]. The results of this systematic literature review show critical findings and lessons learned for both healthcare managers and policymakers of health institutions to improve the governance and application of epidemiological models directed to support effective health polices in the presence of mutant viruses, and environmental and societal changes during pandemic crisis.

## 3. Results

### 3.1. Factors determining a high risk for the emergence of new viral agents

#### 3.1.1. Surveillance of wildlife to avoid spillover effects with emergence and diffusion of new viral agents in humans

Daszak et al. [44] argue some risk factors determining the emergence of new viral agents and pandemic threats, such as the interaction between humas and wildlife that can foster the transmission of dangerous pathogens and spillover effects in human society; a main risk factor is wildlife trade in domestic and international markets that, with poor measures of control, can reduce biosecurity and increase the spillovers leading to new viral agents in humans [45],[46]. In fact, poor surveillance of wildlife can generate evolutionary phases in pathogens that specialize them to transit from animals to humans (cf., Table 2). These factors can trigger compounding and cascading events in regions with a high population density where new viral agents infect a lot of individuals. In addition, persistence of infections, driven by new viral agents, depends on manifold factors, such as: density of population, hygienic conditions, level and period of infectivity in hosts, period necessary for host to achieve a protective immunity, resistance of pathogens to climate and other environmental factors (e.g., air pollution), resistance to pharmaceutical treatments, and continued existence of new viral agents in large regions, albeit extinctions in local clusters, etc. [47].

#### 3.1.2. Biosafety lab risk assessment and protocols to reduce accidents for the emergence and diffusion of new viral agents

Accurate lab risk assessments and security protocols improve biosafety and reduce accidents and the probability that a new viral agent can spread in society [48]. Hellman et al. [49] show that two percent of accidents is in fabrications rooms and thirteen percent is in other places of research labs (Table 2). Van Noorden [50] argues that thirty per cent of people working in labs have assisted with serious injury in a period of about twenty years (see Table 2). Ayi and Ho [51] also report that, in Canada, about fifteen per cent of people in labs have experienced at least one lesion or mishap. Simmons et al. [52] reveal that, at the Iowa State University (U.S.A.), lab incidents are more than eighteen percent of total (Table 2). Kou et al. [53] point out that, at the University of Minnesota (USA) in a period of five years, scholars reported that the most frequent incidents are the spill of hazardous substances, fire and equipment damages that injure lab personnel. These case studies reveal that the escape and diffusion of new viral agents, associated with an accident of lab, can have a vital role in the emergence of epidemics and pandemics [12]. The reduction of incidents in labs that study new viral agents is associated with an accurate activity of lab risk assessments and effective protocols for biosecurity. Li Na et al. [54] argue that risk assessment and biosafety in labs can be performed with different methods, such as scenario analysis, pre-hazard analysis, hazard and operability analysis, fault tree analysis, event tree analysis, matrix analysis, risk mapping, etc. However, control measures of biological risks have no fixed modes generalizable for all labs across different nations, but they have to be adapted to the specificity of lab and country [22],[28]. Moreover, information and data of lab accidents should be linked to a national and international surveillance system for a real time transmission of biological risks in order to better coordinate national security with targeted investigations, and timely interventions in the presence of specific threats and risk for the local and global community [55]. Hence, biosafety risk analyses and risk assessments in laboratories have to be a recurring activity to improve the security of operation and minimize the incidents involving hazardous pathogens and/or aerosol exposure risk to hazardous viral agents that can lead to the emergence of new infectious diseases and serious epidemic and/or pandemic threats at local and global levels [54],[56].

**Table 2.** Average level of incidents in specific labs of universities.

Type and percentage of incidents in labs	Sources
2% of accidents in fabrications rooms 13% in other places of research labs	Hellman et al. [49]
30% of people working in labs has assisted with serious injury over a period of 20 years	Van Noorden [50]
15% of people in labs has experienced at least one lesion or mishap	Ayi and Ho [51]
18% of lab incidents have occurred at the Iowa State University	Simmons et al. [52]
25% of hazard types are spills and fires 18% of hazard types are for equipment failure or explosion 16% of hazard types are associated with cryogenics 15% of hazard types are spills and sharps 12% of serious incidents has involved a researcher requesting an external help
12% is “near miss” incidents	Kou et al. [53]
A natural zoonosis is a rare event	Coccia [12]

#### 3.1.3. High air and environmental pollution, and (*un*)sustainable environment can support pandemic emergence and rapid spread

**Figure 1.**
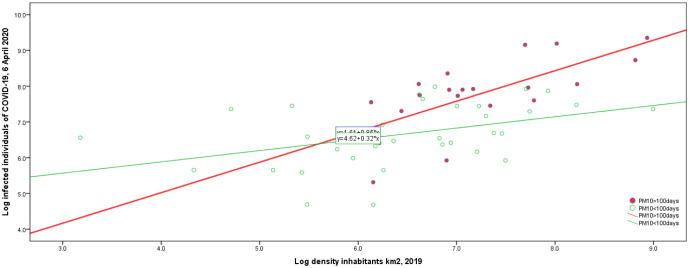
Infected people on population density per km^2^, considering the groups of cities with high or low air pollution (high particulate compounds emissions in the atmosphere). *Note*: Coccia [59] reveals that diffusion of COVID-19 is higher in cities with high air pollution.

One of the factors determining a high risk for the diffusion of epidemics and pandemics similar to COVID-19 is air and environmental pollution in populated cities [11],[57]–[61]. In general, populations living in environments with high air pollution have experienced an increased mortality of COVID-19, because of rapid diffusion of SARS-CoV-2 in polluted and highly populated cities [4,6; cf. Figure 1]. In fact, high levels of particulate matter and other air pollutants can mix with new pathogens, generating mutations and resistance of these new viral agents that increase their transmissibility and infectivity with a negative impact on the health of people [2],[7],[62]–[65].

### 3.2. Pre-emptively measures of control for reducing widespread diffusion of new viral agents

#### 3.2.1. Strengthening the early warning system with effective contract tracing system

**Figure 2.**
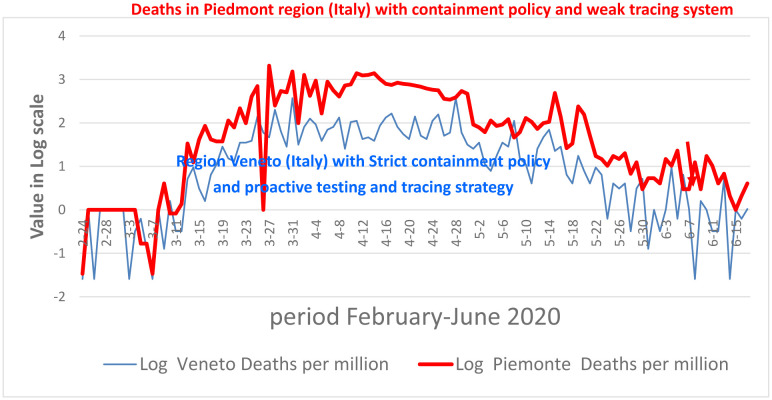
Trends of COVID-19 deaths in Italian regions (Veneto and Piemonte) with effective and non-effective contract tracing systems. *Source*: Benati and Coccia [66].

An effective contact tracing system and timely isolation can reduce transmission dynamics of infectious diseases within, and between, different outbreak areas [66]. This dual strategy plays a basic role to stop the spread of infectious diseases having a latent pre-symptomatic phase (Coccia, 2020). Moreover, an effective contact tracing system, with a “bidirectional” approach, can reduce the spread of new viral agents in society, and improve the timely healthcare in infected individuals to reduce severe side effects and also mortality [67],[68]. Benati and Coccia [66] show that, during the first wave of the COVID-19 pandemic, some regions in Italy have managed pandemic crisis with appropriate health policy responses based on: a) a timely and widespread testing of individuals, b) effective units of epidemiological investigation in a pervasive contact-tracing system to detect and isolate all infected people. Benati and Coccia [66] reveal that widespread and in-depth testing of symptomatic and asymptomatic individuals, associated with a timely isolation of infected people, can reduce total number of infections and deaths of COVID-19 (Figure 2). Hence, a health policy of timely effective contact tracing is basic to face pandemics when there are no effective pharmaceutical treatments, such as vaccines and/or appropriate antiviral drugs [66],[69]. This evidence by Benati and Coccia [66], in the first pandemic wave of COVID-19, provides important lessons when appropriate drugs are not ready, and to design health policies based on effective contact tracing systems can constraint pandemic waves driven by new viral agents or their variants and sub-variants (Figure 2). Zhan et al. [70] also argue that a susceptible-unconfirmed-confirmed-recovered mode can capture transmission dynamics of confirmed cases, and that by increasing five times the testing capacity associated with control measures, the numbers of COVID-19 infections in people would decrease to 33%.

#### 3.2.2. Effective public governance improves prevention and preparedness to face pandemic threats

**Figure 3.**
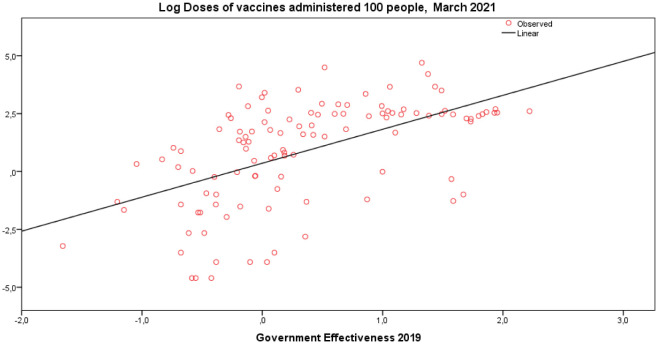
How the increase of government effectiveness between countries can support higher levels of vaccination. *Source*: Benati and Coccia [96].

Effective governance and constant investments in the health sector can support prevention and preparedness to pandemic threats with an improvement of: surveillance in the interaction between human society and wildlife, protocols for biosafety laboratory risk assessments, overall health system, human resources and management in healthcare, new technology that reduces human exposure to new vital agents or that improves treatments of new infections, early warning system and containment actions to stop rapid diffusion of viral agents in cities. In short, good institutions and effective public governance associated with human resources having expertise and availability of new technology in health sector, can improve the preparedness of crisis management to face novel viral agents and consequential pandemic crisis [71]–[95]. Benati and Coccia [96] show the positive effects of good governance for designing effective policy responses to cope with the COVID-19 pandemic, such as the roll out of a timely vaccination plan directed to reduce negative pandemic impact in society (Figure 3).

**Figure 4.**
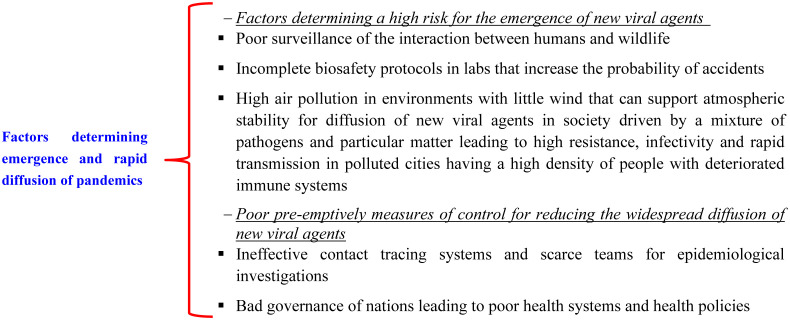
Factors that increase the opportunities for a pandemic virus to emerge and spread.

Figure 4 summarized critical risk factors for the emergence and diffusion of pandemics.

#### 3.2.3. Prediction Models for COVID-19

Pandemic forecasting plays a vital role to control the pandemic threats and support health policies to cope with on-going pandemic crisis [97],[98]. In the presence of the COVID-19 pandemic crisis, scholars suggest different models for epidemic tracking and forecasting [35]. Reinhart et al. [99] have done many efforts in the construction and maintenance of an open repository of real-time and geographically detailed COVID-19 indicators in the United States. This repository provides main information about COVID-19, such as confirmed cases, hospitalizations, intensive care units, deaths, fatality rates, etc. McDonald et al. [100] endeavor to explain if a set of indicators can improve the accuracy of COVID-19 forecasting in the short run across regions. KhudaBukhsh et al. [101] analyze the support that The Ohio State University offered, in the initial wave of COVID-19, to the Ohio Department of Health with epidemic modeling and decision analytics directed to predict statewide cases of new infections, as well as potential hospital burdens in the state. The proposed Dynamical Survival Analysis (DSA)-based statistical method, for statewide prediction and uncertainty quantification, needs fewer parameters and is less computationally expensive than agent-based models. However, the DSA method does not provide the flexibility to test arbitrary what-if scenarios involving individual human behaviors, because the method is based on population-level equations. Hurford et al. [102] analyze an elimination strategy, enacting strict border control and periods of lockdowns to end community transmission. A case study in Canada has reported a long period with no community cases. They also develop a method to assess alternative plans to relax public health restrictions when vaccine coverage is high in regions that have implemented an elimination strategy. Khairulbahri [103] suggests a SEIR model capturing the roles of behavioral measures, partial lockdowns, hospital preparedness and asymptomatic cases in Sweden. The suggested SEIR model successfully reproduces main observed outputs and finds that the effects of partial lockdowns effectively start more than 50 days after the first confirmed case. Hence, behavioral measures and partial lockdowns can reduce infected cases of about 22% and 70%, respectively. Sasanami et al. [104] endeavor to estimate in Japan the proportion of the population that is immune to symptomatic SARS-CoV-2 infection with the Omicron variant (immune proportion), considering the waning of immunity resulting from vaccination and naturally acquired infection. Results show that vaccine-induced immunity, conferred by the second vaccine dose, was estimated to rapidly wane. The resulting prediction of the share of the population that is immune to symptomatic SARS-CoV-2 infection could aid decision-making processes on when, and for whom, another round of booster vaccination should be considered. Li et al. [105] develop a new transmission model *via* a delay differential system, which parameterized the roles of adaptive behaviors and vaccination, allowing to simulate the dynamic infection process among people. Results show that for complete prevention, the average proportions of people with immunity should be about 76–92% with adaptive protection behaviors, or roughly 77–97% without protection behaviors; in addition, the required proportion of vaccinated people is a sub-linear decreasing function of vaccine efficiency, with little heterogeneity between different countries.

Vasconcelos et al. [106] apply a generalized pathway model, with time-dependent parameters, to describe the mortality curves of the COVID-19 for several countries that exhibit multiple waves of infections. The model is in good agreement with the data for selected cases under study, showing the starting and peak dates for each successive wave. In fact, reliable estimates of characteristic points in epidemic curves play important aspects for assessing the effectiveness of interventions, and the possible negative impact of their relaxation. The study also shows that expected time for an epidemic wave to reach a peak seems to be positively correlated with the delay to adopt control measures. The study describes efficiency COVID-19 data, but a more detailed analysis about the effectiveness of intervention measures is needed to improve predictions. Cherednik [107] proposes a two-phase solution for modeling the total number of confirmed cases in COVID-19, and for describing the curves of many pandemic waves. A suggested approach shows uniformity of the COVID-19 waves between countries, and this result can be used for forecasting the epidemic spread. This approach is very different from the classical SID, SIR, SIER models and their variants, and the models used in the neighboring directions of invasion ecology. The saturation in the model is a dynamic equilibrium between the virus invasion and protective measures, but this is not due to classical herd immunity. Moreover, because of a very limited number of parameters in a two-phase solution, the modeling is rigid. Since the forecasting of waves can be potentially reduced to finding the factors of initial transmission rate and the intensity of hard protective measures, the challenge is to do this at early stages of the pandemic waves; likely it is doable near to the turning point of the wave. Currently, these parameters are found “manually”. Leonov et al. [108] consider two modifications to the well-known SEIR model of epidemic development to analyze the infection curves in COVID-19 pandemic. These models can identify external and hidden sources of infection. Numerical experiments show that infection curves can be approximated with a high accuracy (2–8%). However, in infection waves having a sharp increase, the approximation errors grow tenfold. This problem is solved with an additional term that considers external and latent sources of infection (average accuracy here is 2-4%). Moreover, because of lack of data, models approximated the infection waves, including the Omicron wave, with an accuracy of about 10–30%. Cao et al. [109], using a model, show in a *pre*-vaccine era that policy-related factors guide the spread of COVID-19; in the *post*-vaccine era, the drivers of policy-related factors decreased, whereas travel-related factors, variants and vaccine-related factors increased the COVID-19 diffusion. However, a quantitative assessment of the combinatorial effect of different factors is needed to effective control the diffusion of COVID-19 over time. Namiki and Yano [110] use the total number of individuals as an optimized parameter in the susceptible-infected-quarantined-recovery (SIQR) model, and propose two methods to simulate multiple epidemic waves (MEWs). Numerical results indicate that a logistic model fits MEWs with a good accuracy. A limitation of the model is the transition from the delta to omicron variant that generates difficulty of prediction because the rate of the infection changes during the epidemic wave. Safaie et al. [111] analyze the factors that affect COVID-19 outbreak and expand the basic SEIR model by considering vaccination policy. Results suggest that the vital factor that reduces the COVID-19 diffusion is due to increasing vaccination plans, rather than decreasing in behavioral risks of people.

**Table 3.** Limitations of prediction models for COVID-19.

Weaknesses	Authors
Dynamical Survival Analysis-based statistical method does not have the flexibility to test arbitrary what-if scenarios involving individual human behaviors because the method is based on population-level equations	KhudaBukhsh et al. [101]
The pathway approach for multi-wave epidemics shows its efficiency in describing COVID-19 data, but a more detailed study about the effectiveness of intervention measures is needed to improve predictions	Vasconcelos et al. [106]
The modeling is rigid for limited number of parameters in two-phase solution. The key factors of the initial transmission rate and the intensity of hard protective measures are found “manually”	Cherednik [107]
The approximation errors grow tenfold in pandemic waves with a sharp increase of infections. Because of the lack of data, model approximates the infection curves with an accuracy of about 10–30%	Leonov et al. [108]
A quantitative assessment of the combinatorial effect of different control measures is needed for accurate prediction of the diffusion of COVID-19	Cao et al. [109]
Limitation of the model is the transition from the delta to omicron variant because the rate of the infection changes over time in the epidemic wave	Namiki and Yano [110]
Human behavior and its impact on the progression of epidemics are hard to measure and to model	Mohammadi et al. [117]
Unlike predictions of epidemiologic models, the reduction of control measures for COVID-19 did not generate a rapid take off of infections	Wieland [112]
Epidemiologic models for COVID-19 generate overestimation of deaths	Douglas [113]
A limitation of models for pandemic prediction is the assumption of a constant reproductive number, whereas in real contexts it changes over the course of time	Korolev [115]
A lot of models do not consider that the deaths of COVID-19 have a skewed distribution towards elderly and susceptible people with comorbidities	Chen et al. [116]
A critical limitation of epidemiological models for COVID-19 is that they do not consider the behavioral change of people in the presence of a pandemic	Stangier et al. [118]

#### 3.2.4. Limitations in epidemiologic model of forecasting

Different pandemic tracking and forecasting models for COVID-19 crisis support processes of decision-making, but they also have limitations (Table 3). For instance, COVID-19 diffusion has shown rapid peaks and then infections abruptly fall, regardless of measures of control, generating a flop in many prediction modelling. In fact, unlike predictions of different epidemiologic models, the reduction of control measures for COVID-19 did not generate a rapid take off of infections [112]. Other main problems of epidemiologic models for COVID-19 prediction are due to high overestimation of deaths [113]–[115]. Additionally, a limitation of current models for pandemic prediction is the assumption of a constant reproductive number, whereas, in real contexts, it changes over the course of time [115]. A lot of models also do not consider that the deaths of COVID-19 have a skewed distribution towards elderly and susceptible people with comorbidities [116]. As a consequence, appropriate prediction models of COVID-19 should include age-dependent factors.

Another critical limitation of epidemiological models for COVID-19 forecasting is that they did not consider the behavioral change of people in the presence of a pandemic [117]. In fact, people with the fear to be infected and/or of die with a new infection, they adapt the behaviour to new situation taking provident actions to protect themselves and survive in uncertain environments [118]. A main example is in the US economy where the reduction of consumer mobility is due to mainly to private responses rather than public obligations [119]. Finally, susceptible-infectious-removed (SIR), Susceptible-Exposed-Infected-Recovered-Dead (SEIRD) and other epidemiologic models focus mainly on stable and short-run variables, whereas factors driving the pandemic waves have dynamic change over time. However, Amaro [120] argues that, in a small number of selected countries, the SIR model well describes a basic reproduction number between 3 and 8 units. Overall, epidemiologic models are useful approaches for approximate pandemic dynamics but they cannot provide reliable long-run forecasting for rapid changes in manifold factors, such as new variants, relaxation of containment measures, behavioral change, etc. (Table 3).

## 4. Discussion and policy implications for preventing pandemic threats

Studies of literature review on COVID-19 pandemic crisis have focused on different aspects. Noteboom et al. [121], with a literature review, argue that deep learning methods, such as medical image screening, can improve diagnosis of COVID-19, in particular, the application of transfer learning for chest X-rays and computed tomography. Jordan et al. [122] analyze, with a literature review, optimization and machine learning approaches related to aspects of prediction and control of COVID-19 pandemic, such as optimization in screening testing strategies, healthcare resource management, vaccination prioritization, etc. Shakeel et al. [42] present a systematic literature review with results that are vital both for healthcare managers and for prediction model developers to face COVID-19 pandemic. Hudda et al. [123] show a systematic review of COVID-19 prediction models based on preprint and peer-reviewed published manuscripts, in order to assess the percentage of adherence between prediction and effective data. Instead, Zhang et al. [124] systematically examine, in COVID-19 research, the application of three simulation approaches given by a system dynamics model, agent-based model and discrete event simulation, and their hybrids. Results suggest that hybrid simulation models can capture the complexity of pandemic impact to design appropriate interventions of health policy. Bhatia and Di Ruggiero [125] review publicly accessible documents of the Canadian Government about the procurement and provision of expert-driven science advice to support timely decision-making processes of public health for pandemic crisis. The study clarifies that the federal science advice bodies and mechanisms represent main elements of the federal science advice “ecosystem”. Moreover, the study suggests, in the presence of pandemic crisis, the necessity in Canada to institutionalize science advisory bodies for public health to improve pandemic preparedness, and ensure rapid mobilization of well-coordinated and independent advice.

This study extends this body of knowledge by discussing factors that may trigger pandemic threats and the role of pandemic modelling with *pros* and *cons* aspects. In particular, systematic review here reveals that epidemiologic models for the prediction of COVID-19 have also many limitations because of unpredictable dynamics and rapid change in society of the new viral agent SARS-CoV-2 and its variants and sub-variants. This study also provides, considering the analysis done, some recommendations to improve crisis management of pandemics by focusing on key components described in Table 4.

**Table 4.** Elements for improving crisis management of pandemic threats.

ELECTRONIC MEDICAL RECORDS (EMR)
The accurate analytics of real-time data can improve the forecasting of pandemic dynamics. *Electronic Medical Records* (EMR) has a high potential for real-time surveillance streams of infections and deaths related to pandemic of new viral agents similar to SARS-CoV-2
DIFFERENT PHASES OF EPIDEMIC SURVEILLANCE NEED DIFFERENT ANALYTIC TECHNIQUES AND APPROACH.
*In the inter-pandemic phase*, the monitoring of data streams and consequential events worldwide can avoid compounding and cascading events, such as species jumping, mutations and high diffusion of viral agents. *In the containment phase*, a threat of a new viral agent has to be intensely monitored, assessed, and contained with effective contact tracing systems. A real-time analytics can provide reliable estimation of critical epidemiological parameters for assessing and predicting with accuracy pandemic trend, and controlling diffusion with appropriate health policies.

In general, countries should direct their efforts on pre-emptively strategic actions to increase R&D investments in new technology, organized infrastructures in health sector, equipment, and education of human resources, associated with international collaboration, for improving activities of prevention and preparedness to cope timely with unforeseen pandemics and reduce problems of public health [18],[22],[28]. In particular, preventive strategies should minimize risk factors associated with the emergence and evolution of a new pandemic virus [126]–[128].

This strategy of pandemic prevention should focus on following vital guidelines:

- Analytics for detecting factors determining pandemic threat.
- Reduction of factors associated with emergence and diffusion of new viral agents to prevent pandemics.
- Health policies that mitigate negative impact of outbreaks in society, endeavoring to contain and/or stop diffusion in local contexts.

This strategy can be implemented with three main actions.

First, the reduction of interaction between humans and wildlife and/or appropriate protections in hazardous environments (e.g., mines) for reducing human exposure to wild animals inducing a high risk that a pandemic virus to emerge (e.g., spillover from bats, rats, etc.).

Second, the improvement of the warning system and prompt containment interventions in the initial phase of an outbreak that could prevent chains of transmission in local and global contexts. An effective early warning system in the international community can ensure timely detection of suspected cases in humans. Laboratories have to receive all data, information and clinical specimens for assessing risk factors of a pandemic threat in society and communicate timely appropriate actions to remove and/or minimize factors for a diffusion from local to global environments, such as selected restrictions in specific places, also considering that asymptomatic people may not be infectious for the surrounding people [17],[129]–[131]. International institutions have to timely coordinate global health policies to minimize risks of pandemic threat or global crisis.

Third, governments have to support public and private research labs for drug discoveries of effective antivirals and/or vaccines to treat new viral agents [28]. These innovative drugs to face health emergency should be delivered to all countries with equity to reduce the takeoff of epidemic/pandemic in local and/or global contexts and the generation of new variants that feed the evolution of pandemic waves for a longer period of time [8],[28],[34],[66],[96],[132]. R&D investments should also be directed to new vaccines that provide a general and long-run protection of people against mutant viral agents that generate variants and sub-variants [2],[16],[18],[19],[133],[134].

Overall, then, R&D investments and good governance in countries can improve the preparedness and reduce opportunities of human exposure to hazardous pathogens and risk factors that lead to the emergence of a pandemic virus, strengthening the early warning system that decreases/stops transmissibility among humans in the initial phase, and also delays its international spread [2],[18],[28].

## 5. Conclusions

Lessons learned from the COVID-19 pandemic crisis suggest that governments have to plan strategies and policies of public health to prevent and prepare countries to cope with future infectious diseases of new vital agents in society [81],[135]–[137]. In a worldwide context, an effective crisis management is based, more and more, on an international collaboration in science and public health for timely sharing of data and samples for accurate analytics of novel viral agents in order to apply appropriate health policy responses that contain hazardous pathogens in local communities and avoid the spread between countries [2]. Considering the difficulties of accurate long-run outlook of pandemic threats, as well as of exact predictions of on-going pandemic trends because of manifold factors that change rapidly, countries have to focus their efforts on planning flexible and resilient strategies that prevent the emergence and diffusion of a pandemic virus from local to global environments [28],[66],[96]. As far as the current evolution of COVID-19 in 2023, after almost three years of the health emergence, El-Sadr et al. [15] argue a need for unbiased monitoring of transmission and infection rates by means of regular testing of sentinel populations or randomly selected representative samples of the general population [138]. Moreover, vaccine and booster coverage, availability and utilization of new treatment drugs for COVID-19 are critical variables that affect the risk of severe illness in population, numbers of deaths from SARS-CoV-2 and its variants, and health system capacity to support a huge burden in a short period. Vaccination plan can avoid negative socioeconomic effects driven by unintended results of mitigation measures to face pandemics, such as stay-at-home orders, the shutting down of public venues, ban travels, etc. Government and institutional entities need also create clear pathways for vulnerable and poor people [139]. One of the key challenges, according to El-Sadr et al. [15], is that public health policies should move away, as the pandemic evolves, from universal and general population-wide prevention policies to differentiated measures considering characteristics of various communities, places and also the evolution of novel viral agents in the environment and society [140]. In short, as the pandemic evolves, the control measures and their effects are more complex, and clear guidelines should be applied to specific populations, leveraging an effective communication that explains the rationale for various recommendations. The post-(COVID-19) pandemic period needs to avoid using alarming and misleading language in order to suggest, with effective communication, reasonable solutions to bring people with a health security plans towards a new nonemergency phase of the pandemic.

Overall, the preparation and crisis management to health emergencies have to be planned by countries with forward-looking policies that improve institutions, public governance and communication in health sectors and not with short-run interventions prepared for pre-, on-going, and post pandemic scenarios [141]–[145].

Although this study has described interesting lessons and insights that can support to face future pandemics, results are, of course, tentative.

One of the problems is the difficulty of an accurate prediction of the numbers of reported and unreported cases for the COVID-19 pandemic, and similar pandemics for different age classes [146]. Second, the change of Reproduction number in the presence of rapid evolutionary changes of viral agents in variants and sub-variants should be considered to improve the accurateness of epidemiologic modelling of prediction [87],[147],[148]. Finally, a lot of confounding and situational factors should be considered for designing accurate measures of preparedness and prediction for future pandemics. In fact, the Global Health Security Index that assesses the preparedness of countries to face a biological threat ranked, in 2019, the United States of America and the UK as first and second place, respectively, suggesting a strong capability of these countries to face a major biological threat [150],[151]–[174]. Instead, these countries have experienced a current fatality rate of 1.08% for the USA and 0.89% for the UK, respectively, which is higher than other advanced countries, such as France and Germany.

Hence, to conclude, prevention, crisis management of pandemics and forecasting of the dynamics of novel viral agents is a difficult task in a more and more turbulent world, though we have considerable scientific and technological advances.

## References

[1] Chowdhury T, Chowdhury H, Bontempi E (2022). Are mega-events super spreaders of infectious diseases similar to COVID-19? A look into Tokyo 2020 Olympics and Paralympics to improve preparedness of next international events. Environ Sci Pollut R.

[2] Coccia M (2020). Factors determining the diffusion of COVID-19 and suggested strategy to prevent future accelerated viral infectivity similar to COVID. Sci Total Environ.

[3] Coccia M (2020). Two mechanisms for accelerated diffusion of COVID-19 outbreaks in regions with high intensity of population and polluting industrialization: the air pollution-to-human and human-to-human transmission dynamics. MedRxiv.

[4] Coccia M (2020). Effects of Air Pollution on COVID-19 and Public Health, Research Article-Environmental Economics-Environmental Policy. ResearchSquare.

[5] Coccia M (2021). Recurring waves of Covid-19 pandemic with different effects in public health. J Econ Bib.

[6] Coccia M (2021). High health expenditures and low exposure of population to air pollution as critical factors that can reduce fatality rate in COVID-19 pandemic crisis: a global analysis. Environ Res.

[7] Coccia M (2021). Effects of the spread of COVID-19 on public health of polluted cities: results of the first wave for explaining the *dejà vu* in the second wave of COVID-19 pandemic and epidemics of future vital agents. Environ Sci Pollut R.

[8] Coccia M (2022). COVID-19 pandemic over 2020 (with lockdowns) and 2021 (with vaccinations): similar effects for seasonality and environmental factors. Environ Res.

[9] Bontempi E, Coccia M (2021). International trade as critical parameter of COVID-19 spread that outclasses demographic, economic, environmental, and pollution factors. Environ Res.

[10] Bontempi E, Coccia M, Vergalli S (2021). Can commercial trade represent the main indicator of the COVID-19 diffusion due to human-to-human interactions? A comparative analysis between Italy, France, and Spain. Environ Res.

[11] Akan AP, Coccia M (2022). Changes of Air Pollution between Countries Because of Lockdowns to Face COVID-19 Pandemic. Appl Sci.

[12] Coccia M (2022). Meta-analysis to explain unknown causes of the origins of SARS-COV-2. Environ Res.

[13] Johns Hopkins Center for System Science and Engineering (2022). Coronavirus COVID-19 Global Cases.

[14] Núñez-Delgado A, Bontempi E, Coccia M (2021). SARS-CoV-2 and other pathogenic microorganisms in the environment. Environ Res.

[15] El-Sadr Wafaa M, Ashwin Vasan, Ayman El-Mohande (2023). Facing the New Covid-19 Reality. N Engl J Med.

[16] Coccia M (2023). Effects of strict containment policies on COVID-19 pandemic crisis: lessons to cope with next pandemic impacts. Environ Sci Pollut R.

[17] Coccia M (2021). The relation between length of lockdown, numbers of infected people and deaths of COVID-19, and economic growth of countries: Lessons learned to cope with future pandemics similar to COVID-19. Sci Total Environ.

[18] Coccia M (2022). Improving preparedness for next pandemics: Max level of COVID-19 vaccinations without social impositions to design effective health policy and avoid flawed democracies. Environ Res.

[19] Coccia M (2022). COVID-19 Vaccination is not a Sufficient Public Policy to face Crisis Management of next Pandemic Threats. Publc Organ Rev.

[20] Farazmand A (2001). Handbook of crisis and emergency management.

[21] Farazmand A (2014). Crisis and Emergency Management, Theory and Practice.

[22] Coccia M (2022). Preparedness of countries to face covid-19 pandemic crisis: Strategic positioning and underlying structural factors to support strategies of prevention of pandemic threats. Environ Res.

[23] Dai H, Cao W, Tong X (2022). Global prediction model for COVID-19 pandemic with the characteristics of the multiple peaks and local fluctuations. BMC Med Res Methodol.

[24] Krechetov M, Esmaieeli Sikaroudi AM, Efrat A (2022). Prediction and prevention of pandemics via graphical model inference and convex programming. Sci Rep.

[25] Kuvvetli Y, Deveci M, Paksoy T (2021). A predictive analytics model for COVID-19 pandemic using artificial neural networks. Decis Anal J.

[26] Liu Z, Magal P, Webb G (2021). Predicting the number of reported and unreported cases for the COVID-19 epidemics in China, South Korea, Italy, France, Germany and United Kingdom. J Theor Biol.

[27] Šušteršič T, Blagojević A, Cvetković D (2021). Epidemiological Predictive Modeling of COVID-19 Infection: Development, Testing, and Implementation on the Population of the Benelux Union. Front Public Health.

[28] Coccia M (2021). Pandemic Prevention: Lessons from COVID-19. Encyclopedia.

[29] Khandia R, Singhal S, Alqahtani T (2022). Emergence of SARS-CoV-2 Omicron (B.1.1.529) variant, salient features, high global health concerns and strategies to counter it amid ongoing COVID-19 pandemic. Environ Res.

[30] Groh M (2014). Strategic Management in Times of Crisis. Am J Econ Sociol Bus Adm.

[31] Alsobh A (2022). Prediction of COVID-19 Disease by ARIMA Model and Tuning Hyperparameter Through GridSearchCV. Emerging Technologies in Data Mining and Information Security: Proceedings of IEMIS.

[32] Keshavamurthy R, Dixon S, Pazdernik KT (2022). Predicting infectious disease for biopreparedness and response: A systematic review of machine learning and deep learning approaches. One Health.

[33] Magazzino C, Mele M, Coccia M (2022). A machine learning algorithm to analyze the effects of vaccination on COVID-19 mortality. Epidemiol Infect.

[34] Coccia M (2022). Optimal levels of vaccination to reduce COVID-19 infected individuals and deaths: A global analysis. Environ Res.

[35] Rosenfeld R, Tibshirani RJ (2021). Epidemic tracking and forecasting: Lessons learned from a tumultuous year. PNAS.

[36] Collins GS, Ma J, Dhiman P (2021). There are no shortcuts in the development and validation of a COVID-19 prediction model. Transbound Emerg Dis.

[37] PubMed (2023). Search.

[38] Scopus (2023). Strart exploring.

[39] Web of Science (2023). Documents.

[40] Herby J, Jonung L, Hanke SH (2022). A literature review and meta-analysis of the effects of lockdowns on COVID-19 mortality. Stud Appl Econ.

[41] Petticrew M, Roberts H (2005). Systematic reviews in the social sciences: A practical guide.

[42] Shakeel SM, Kumar NS, Madalli PP (2021). Covid-19 prediction models: A systematic literature review. Osong Public Health and Research Perspectives.

[43] Clarke J (2011). What is a systematic review?. Evid-Based Nu.

[44] Daszak P, Olival KJ, Li H (2020). A strategy to prevent future epidemics similar to the 2019-nCoV outbreak. Biosafety and Health.

[45] Anderson RM, May RM (1991). Infectious Diseases of Humans: Dynamics and Control.

[46] Dobson AP, Carper ER (1996). Infectious diseases and human population history. Bioscience.

[47] Wolfe N, Dunavan C, Diamond J (2007). Origins of major human infectious diseases. Nature.

[48] Ménard AD, Trant JF (2020). A review and critique of academic lab safety research. Nat Chem.

[49] Hellman MA, Savage EP, Keefe TJ (1986). Epidemiology of accidents in academic chemistry laboratories. J Chem Educ.

[50] Van Noorden R (2013). Safety survey reveals lab risks. Nature.

[51] Ayi HR, Hon CY (2018). Safety culture and safety compliance in academic laboratories: A Canadian perspective. J Chem. Health Saf.

[52] Simmons HE, Matos B, Simpson SA (2017). Analysis of injury data to improve safety and training. J Chem Health Saf.

[53] Kou Y, Peng X, Dingwell CE (2021). Learning experience reports improve. Acad Res Saf J Che Educ.

[54] Li Na, Hu LF, Jin AJ (2019). Biosafety laboratory risk assessment. J Bios Biosecur.

[55] Jia P, Yang SJ (2020). China needs a national intelligent syndromic surveillance system. Nature Med.

[56] Yuan D, Gao W, Liang S (2020). Biosafety threats of the rapidly established labs for SARS-CoV-2 tests in China. Environ Int.

[57] Coccia M (2020). An index to quantify environmental risk of exposure to future epidemics of the COVID-19 and similar viral agents: Theory and Practice. Environ Res.

[58] Coccia M (2020). How (Un)sustainable Environments are Related to the Diffusion of COVID-19: The Relation between Coronavirus Disease 2019, Air Pollution, Wind Resource and Energy. Sustainability.

[59] Coccia M (2021). How do low wind speeds and high levels of air pollution support the spread of COVID-19?. Atmos Pollut Res.

[60] Coccia M (2021). The effects of atmospheric stability with low wind speed and of air pollution on the accelerated transmission dynamics of COVID-19. International Journal of Environmental Studies.

[61] Coccia M, Bellitto M (2018). A critique of human progress: a new definition and inconsistencies in society. Quaderni IRCrES-CNR.

[62] Jones AM, Harrison RM (2004). The effects of meteorological factors on atmospheric bio aerosol concentrations-a review. Sci Total Environ.

[63] Wei M, Liu H, Chen J (2020). Effects of aerosol pollution on PM2.5-associated bacteria in typical inland and coastal cities of northern China during the winter heating season. Environ pollut.

[64] Zhong J, Zhang X, Dong Y (2018). Feedback effects of boundary-layer meteorological factors on explosive growth of PM2.5 during winter heavy pollution episodes in Beijing from 2013 to 2016. Atmos Chem Phys.

[65] Rosario DKA, Mutz YS, Bernardes PC (2020). Relationship between COVID-19 and weather: Case study in a tropical country. Int J Hyg Envir Heal.

[66] Benati I, Coccia M (2022). Effective Contact Tracing System Minimizes COVID-19 Related Infections and Deaths: Policy Lessons to Reduce the Impact of Future Pandemic Diseases. J Public Admin Gov.

[67] Bradshaw WJ, Alley EC, Huggins JH (2021). Bidirectional contact tracing could dramatically improve COVID-19 control. Nat Commun.

[68] Yalaman A, Basbug G, Elgin C (2021). Cross-country evidence on the association between contact tracing and COVID-19 case fatality rates. Sci Rep-UK.

[69] Coccia M, Benati I (2018). Rewards in public administration: A proposed classification. J Soc Adm Sci.

[70] Zhan C, Chen J (2021). An investigation of testing capacity for evaluating and modeling the spread of coronavirus disease. Inform Sciences.

[71] Coccia M (2012). Political economy of R&D to support the modern competitiveness of nations and determinants of economic optimization and inertia. Technovation.

[72] Coccia M (2014). Driving forces of technological change: The relation between population growth and technological innovation-Analysis of the optimal interaction across countries. Technol Forecast Soc.

[73] Coccia M (2018). Classification of innovation considering technological interaction. J Econ Bib.

[74] Coccia M (2018). General properties of the evolution of research fields: a scientometric study of human microbiome, evolutionary robotics and astrobiology. Scientometrics.

[75] Coccia M (2018). Types of government and innovative performance of countries. J Soc Adm Sci.

[76] Coccia M (2019). Comparative Institutional Changes. Global Encyclopedia of Public Administration, Public Policy, and Governance.

[77] Coccia M (2019). Intrinsic and extrinsic incentives to support motivation and performance of public organizations. J Econ Biblio.

[78] Coccia M (2019). Metabolism of Public Organizations. Global Encyclopedia of Public Administration, Public Policy, and Governance.

[79] Coccia M (2020). Fishbone diagram for technological analysis and foresight. Int J Foresight and Innovation Policy.

[80] Coccia M (2020). The evolution of scientific disciplines in applied sciences: dynamics and empirical properties of experimental physics. Scientometrics.

[81] Coccia M (2021). Comparative Critical Decisions in Management. Global Encyclopedia of Public Administration, Public Policy, and Governance.

[82] Coccia M (2022). Critical innovation strategies for achieving competitive strategic entrepreneurship in ever-increasing turbulent markets. Strategic Entrepreneurship-Perspectives on Dynamics, Theories, and Practices.

[83] Coccia M (2022). Technological trajectories in quantum computing to design a quantum ecosystem for industrial change. Technology Analysis & Strategic Management.

[84] Coccia M, Benati I (2017). What is the relation between public manager compensation and government effectiveness? An explorative analysis with public management implications. Quaderni Ircres-CNR.

[85] Coccia M, Mosleh M, Roshani S (2022). Evolution of quantum computing: Theoretical and innovation management implications for emerging quantum industry. IEEE Transactions on Engineering Management.

[86] Coccia M, Roshani S, Mosleh M (2021). Scientific Developments and New Technological Trajectories in Sensor Research. Sensors.

[87] Coccia M (2019). Revolutions and Evolutions. Global Encyclopedia of Public Administration, Public Policy, and Governance.

[88] Coccia M, Roshani S, Mosleh M (2022). Evolution of Sensor Research for Clarifying the Dynamics and Properties of Future Directions. Sensors.

[89] Mosleh M, Roshani S, Coccia M (2022). Scientific laws of research funding to support citations and diffusion of knowledge in life science. Scientometrics.

[90] Núñez-Delgado Avelino, Zhien Zhang, Elza Bontempi (2023). Editorial on the Topic “New Research on Detection and Removal of Emerging Pollutants”. Materials.

[91] Pagliaro M, Coccia M (2021). How self-determination of scholars outclasses shrinking public research lab budgets, supporting scientific production: a case study and R&D management implications. Heliyon.

[92] Pronti A, Coccia M (2021). Agroecological and conventional agricultural systems: comparative analysis of coffee farms in Brazil for sustainable development. Int J Sustainable Development.

[93] Roshani S, Bagheri R, Mosleh M (2021). What is the relationship between research funding and citation-based performance? A comparative analysis between critical disciplines. Scientometrics.

[94] Roshani S, Coccia M, Mosleh M (2022). Sensor Technology for Opening New Pathways in Diagnosis and Therapeutics of Breast, Lung, Colorectal and Prostate Cancer. HighTech Innov.

[95] Sagan A, Thomas S, McKee M (2020). COVID-19 and health systems resilience: lessons going forwards. Eurohealth.

[96] Benati I, Coccia M (2022). Global analysis of timely COVID-19 vaccinations: Improving governance to reinforce response policies for pandemic crises. Int J Health Gov.

[97] Ajelli M (2018). The RAPIDD Ebola forecasting challenge: Model description and synthetic data generation. Epidemics.

[98] Johansson MA, Apfeldorf KM, Dobson S (2019). An open challenge to advance probabilistic forecasting for dengue epidemics. Proc Natl Acad Sci USA.

[99] Reinhart A, Brooks L, Jahja M (2021). An open repository of real-time COVID-19 indicators. Proc Natl Acad Sci USA.

[100] McDonald DJ (2021). Can auxiliary indicators improve COVID-19 forecasting and hotspot prediction?. Proc Natl Acad Sci USA.

[101] KhudaBukhsh WR, Bastian CD, Wascher M (2023). Projecting COVID-19 cases and hospital burden in Ohio. J Theor Biol.

[102] Hurford A, Martignoni MM, Loredo-Osti JC (2023). Pandemic modelling for regions implementing an elimination strategy. J Theor Biol.

[103] Khairulbahri M (2023). The SEIR model incorporating asymptomatic cases, behavioral measures, and lockdowns: Lesson learned from the COVID-19 flow in Sweden. Biomed Signal Proces.

[104] Sasanami M, Fujimoto M, Kayano T (2023). Projecting the COVID-19 immune landscape in Japan in the presence of waning immunity and booster vaccination. J Theor Biol.

[105] Li Z, Zhao J, Zhou Y (2023). Adaptive behaviors and vaccination on curbing COVID-19 transmission: Modeling simulations in eight countries. J Theor Biol.

[106] Vasconcelos GL, Pessoa NL, Silva NB (2022). Multiple waves of COVID-19: a pathway model approach. Nonlinear Dyn.

[107] Cherednik I (2022). Modeling the Waves of Covid-19. Acta Biotheor.

[108] Leonov A, Nagornov O, Tyuflin S (2023). Modeling of Mechanisms of Wave Formation for COVID-19. Epidemic Math.

[109] Cao Z, Qiu Z, Tang F (2022). Drivers and forecasts of multiple waves of the coronavirus disease 2019 pandemic: A systematic analysis based on an interpretable machine learning framework. Transbound Emerg Dis.

[110] Namiki M, Yano R (2022). A numerical method to calculate multiple epidemic waves in COVID-19 with a realistic total number of people involved. J Stat Mech.

[111] Safaie Nasser, Kaveie Maryam, Mardanian Siroos (2022). Investigation of Factors Affecting COVID-19 and Sixth Wave Management Using a System Dynamics Approach. J Healthc Eng.

[112] Wieland T (2020). A phenomenological approach to assessing the effectiveness of COVID-19 related nonpharmaceutical interventions in Germany. Safety Sci.

[113] Douglas WA (2022). Covid-19 Lockdown Cost/Benefits: A Critical Assessment of the Literature. Int J Econ Bus.

[114] Biggs AT, Littlejohn LF (2021). Revisiting the initial COVID-19 pandemic projections. The Lancet Microbe.

[115] Korolev I (2021). Identification and estimation of the SEIRD epidemic model for COVID-19. J Econometrics.

[116] Chen C, So M, Liu FC (2022). Assessing government policies' impact on the COVID-19 pandemic and elderly deaths in East Asia. Epidemiol Infect.

[117] Mohammadi Z, Cojocaru MG, Thommes EW (2022). Human behaviour, NPI and mobility reduction effects on COVID-19 transmission in different countries of the world. BMC Public Health.

[118] Stangier U, Kananian S, Schüller J (2022). Perceived vulnerability to disease, knowledge about COVID-19, and changes in preventive behavior during lockdown in a German convenience sample. Curr Psychol.

[119] Goolsbee A, Syverson C (2021). Fear, lockdown, and diversion: Comparing drivers of pandemic economic decline 2020. J Public Econ.

[120] Amaro JE (2023). Systematic description of COVID-19 pandemic using exact SIR solutions and Gumbel distributions. Nonlinear Dyn.

[121] Noteboom CB, Zeng D, Godasu R (2021). Applications of deep learning augmented systems for Covid-19 predictions- A literature review.

[122] Jordan E, Shin DE, Leekha S (2021). Optimization in the Context of COVID-19 Prediction and Control: A Literature Review. IEEE Access.

[123] Hudda MT, Archer L, van Smeden M (2023). Minimal reporting improvement after peer review in reports of COVID-19 prediction models: systematic review. J Clin Epidemiol.

[124] Zhang W, Liu S, Osgood N (2023). Using simulation modelling and systems science to help contain COVID-19: A systematic review. Syst Res Behav Sci.

[125] Bhatia D, Allin S, Di Ruggiero E (2023). Mobilization of science advice by the Canadian federal government to support the COVID-19 pandemic response. Hum Soc Sci Commun.

[126] Bundy J, Pfarrer MD, Short CE (2017). Crises and Crisis Management: Integration, Interpretation, and Research Development. J Manage.

[127] Seeger MW, Sellno TL, Ulmer RR (1998). Communication, organization and crisis. Communication Yearbook.

[128] Mahmoudi J, Xiong C (2022). How social distancing, mobility, and preventive policies affect COVID-19 outcomes: Big data-driven evidence from the District of Columbia-Maryland-Virginia (DMV) megaregion. PloS One.

[129] Cao S, Gan Y, Wang C (2020). Post-lockdown SARS-CoV-2 nucleic acid screening in nearly ten million residents of Wuhan, China. Nat Commun.

[130] Tsiotas D, Magafas L (2020). The Effect of Anti-COVID-19 Policies on the Evolution of the Disease: A Complex Network Analysis of the Successful Case of Greece. Physics.

[131] Warren GW, Lofstedt R, Wardman JK (2021). COVID-19: the winter lockdown strategy in five European nations. J Risk Res.

[132] Crow DA, Albright EA, Ely T (2018). Do disasters lead to learning? Financial policy change in local government. Rev Policy Res.

[133] Kapitsinis N (2020). The underlying factors of the COVID-19 spatially uneven spread. Initial evidence from regions in nine EU countries. Reg Sci Policy Pract.

[134] Williams GA, Ulla Díez SM, Figueras J (2020). Translating evidence into policy during the covid-19 pandemic: bridging science and policy (and politics). Eurohealth.

[135] Newby JM, O'Moore K, Tang S (2020). Acute mental health responses during the COVID-19 pandemic in Australia. PloS One.

[136] Sirois FM, Owens J (2021). Factors Associated With Psychological Distress in Health-Care Workers During an Infectious Disease Outbreak: A Rapid Systematic Review of the Evidence. Front Psychiatry.

[137] Whittaker C, Kamaura LT, Takecian PL (2021). Three-quarters attack rate of SARS-CoV-2 in the Brazilian Amazon during a largely unmitigated epidemic. Science.

[138] Christie A, Brooks JT, Hicks LA (2021). Guidance for implementing COVID-19 prevention strategies in the context of varying community transmission levels and vaccination coverage. MMWR Morb Mortal Wkly Rep.

[139] Bleser WK, Shen H, Crook HL (2022). Health policy brief: pandemic-driven health policies to address social needs and health equity.

[140] Overton D, Ramkeesoon SA, Kirkpatrick K (2021). Lessons from the COVID-19 crisis on executing communications and engagement at the community level during a health crisis.

[141] Ackoff RL, Rovin S (2003). Redesigning Society.

[142] Gigerenzer G, Todd PM (1999). Ecological rationality: the normative study of heuristics, Ecological Rationality: Intelligence in the World.

[143] Janssen M, van der Voort H (2020). Agile and adaptive governance in crisis response: Lessons from the COVID-19 pandemic. Int J Inform Manage.

[144] Kahneman D, Slovic P, Tversky A (1982). Judgment Under Uncertainty: Heuristics and Biases.

[145] Weible CM, Nohrstedt D, Cairney P (2020). COVID-19 and the policy sciences: initial reactions and perspectives. Policy Sci.

[146] Liu XX, Fong SJ, Dey N (2021). A new SEAIRD pandemic prediction model with clinical and epidemiological data analysis on COVID-19 outbreak. Appl Intell.

[147] Chen B, Zhao Y, Jin Z (2023). Twice evasions of Omicron variants explain the temporal patterns in six Asian and Oceanic countries. Bmc Infect Dis.

[148] Milanesi S, Rosset F, Colaneri M (2023). Early detection of variants of concern via funnel plots of regional reproduction numbers. Scientific reports.

[149] Cameron EE, Nuzz JR, Bell JA (2019). GHS Index Building. Global Health Security Index. Collective Action and Accountability.

[150] Stribling J, Clifton A, McGill G (2020). Examining the UK Covid-19 mortality paradox: pandemic preparedness, healthcare expenditure, and the nursing workforce. J Adv Nurs.

[151] Coccia M (2019). Comparative World-Systems Theories. Global Encyclopedia of Public Administration, Public Policy, and Governance.

[152] Coccia M (2018). World-System Theory: A sociopolitical approach to explain World economic development in a capitalistic economy. J Econ Pol Econ.

[153] Coccia M (2017). The source and nature of general purpose technologies for supporting next K-waves: Global leadership and the case study of the U.S. Navy's Mobile User Objective System. Technol Forecast Soc.

[154] Coccia M (2015). General sources of general purpose technologies in complex societies: Theory of global leadership-driven innovation, warfare and human development. Technol Soc.

[155] Coccia M (2019). The Role of Superpowers in Conflict Development and Resolutions. Global Encyclopedia of Public Administration, Public Policy, and Governance.

[156] Coccia M (2020). Destructive Technologies for Industrial and Corporate Change. Global Encyclopedia of Public Administration, Public Policy, and Governance.

[157] Coccia M (2019). Theories of Development. Global Encyclopedia of Public Administration, Public Policy, and Governance.

[158] Coccia M (2014). Converging scientific fields and new technological paradigms as main drivers of the division of scientific labour in drug discovery process: the effects on strategic management of the R&D corporate change. Technol Anal Strateg.

[159] Coccia M (2016). Radical innovations as drivers of breakthroughs: characteristics and properties of the management of technology leading to superior organizational performance in the discovery process of R&D labs. Technol Anal Strateg.

[160] Coccia M (2020). Asymmetry of the technological cycle of disruptive innovations. Technol Anal Strateg.

[161] Coccia M (2021). Evolution and structure of research fields driven by crises and environmental threats: the COVID-19 research. Scientometrics.

[162] Coccia M (2019). Metabolism of public research organizations: how do laboratories consume state subsidies?. Publc Organ Rev.

[163] Coccia M (2021). Effects of human progress driven by technological change on physical and mental health. Studi Di Sociologia.

[164] Coccia M (2018). Motivation and theory of self-determination: Some management implications in organizations. J Econ Bib.

[165] Coccia M (2017). New directions in measurement of economic growth, development and under development. J Econ Pol Econ.

[166] Coccia M (2010). Foresight of technological determinants and primary energy resources of future economic long waves. Int J Foresight Innov Policy.

[167] Coccia M (2009). A new approach for measuring and analyzing patterns of regional economic growth: empirical analysis in Italy. A New Approach for Measuring and Analysing Patterns of Regional Economic Growth.

[168] Coccia M, Benati I (2018). Comparative Evaluation Systems. Global Encyclopedia of Public Administration, Public Policy, and Governance.

[169] Coccia M (2021). How a Good Governance of Institutions Can Reduce Poverty and Inequality in Society?. Legal-Economic Institutions, Entrepreneurship, and Management: Perspectives on the Dynamics of Institutional Change from Emerging Markets.

[170] Coccia M (2018). Competition between basic and applied research in the organizational behaviour of public research labs. J Econ Lib.

[171] Coccia M (2018). Economic inequality can generate unhappiness that leads to violent crime in society. Int J Happiness and Development.

[172] Coccia M (2022). Probability of discoveries between research fields to explain scientific and technological change. Technol Soc.

[173] Coccia M (2021). Technological Innovation. The Blackwell Encyclopedia of Sociology.

[174] Coccia M, Benati I (2018). Comparative Studies. Global Encyclopedia of Public Administration, Public Policy, and Governanc.

